# Optimizing microsurgical skills with EEG neurofeedback

**DOI:** 10.1186/1471-2202-10-87

**Published:** 2009-07-24

**Authors:** Tomas Ros, Merrick J Moseley, Philip A Bloom, Larry Benjamin, Lesley A Parkinson, John H Gruzelier

**Affiliations:** 1 Department of Psychology, Goldsmiths, University of London, London, UK; 2 Department of Optometry and Visual Science, City University, London, UK; 3 Western Eye Hospital, London, UK; 4 Department of Ophthalmology, Stoke Mandeville Hospital, Aylesbury, UK; 5 Brainhealth, The Diagnostic Clinic, London, UK

## Abstract

**Background:**

By enabling individuals to self-regulate their brainwave activity in the field of optimal performance in healthy individuals, neurofeedback has been found to improve cognitive and artistic performance. Here we assessed whether two distinct EEG neurofeedback protocols could develop surgical skill, given the important role this skill plays in medicine.

**Results:**

National Health Service trainee ophthalmic microsurgeons (N = 20) were randomly assigned to either Sensory Motor Rhythm-Theta (SMR) or Alpha-Theta (AT) groups, a randomized subset of which were also part of a wait-list 'no-treatment' control group (N = 8). Neurofeedback groups received eight 30-minute sessions of EEG training. Pre-post assessment included a skills lab surgical procedure with timed measures and expert ratings from video-recordings by consultant surgeons, together with state/trait anxiety self-reports. SMR training demonstrated advantages absent in the control group, with improvements in surgical skill according to 1) the expert ratings: overall technique (d = 0.6, p < 0.03) and suture task (d = 0.9, p < 0.02) (judges' intraclass correlation coefficient = 0.85); and 2) with overall time on task (d = 0.5, p = 0.02), while everyday anxiety (trait) decreased (d = 0.5, p < 0.02). Importantly the decrease in surgical task time was strongly associated with SMR EEG training changes (p < 0.01), especially with continued reduction of theta (4–7 Hz) power. AT training produced marginal improvements in technique and overall performance time, which were accompanied by a standard error indicative of large individual differences. Notwithstanding, successful within session elevation of the theta-alpha ratio correlated positively with improvements in overall technique (r = 0.64, p = 0.047).

**Conclusion:**

SMR-Theta neurofeedback training provided significant improvement in surgical technique whilst considerably reducing time on task by 26%. There was also evidence that AT training marginally reduced total surgery time, despite suboptimal training efficacies. Overall, the data set provides encouraging evidence of optimised learning of a complex medical specialty via neurofeedback training.

## Background

The use of EEG biofeedback technology (neurofeedback) to self-regulate brainwave frequencies with the aim of recovering or optimising function and performance is becoming increasingly established. Its clinical applications include the treatment of epilepsy [1], attention hyperactivity disorders [2] and addiction [3]. Meanwhile it has also assumed a role in optimising performance in healthy individuals within fields as diverse as cognition, sport and artistry [4], including NASA research aimed at minimizing pilot error [5]. In particular, recent studies report significant improvements in attention [6,7], memory [8], mental rotation [9], mood [10], dance [11] and musical performance [12].

The set of skills required to undertake surgical and microsurgical procedures includes many of the cognitive and sensorimotor skills which neurofeedback has been shown to enhance. The demands on those undergoing surgical training are considerable and often stressful [13,14]. There may also exist time pressures on those seeking to acquire surgical skills and the availability of expert trainers is often at a premium. To this end there is investment in developing and evaluating procedures to enhance surgical training and performance such as simulation, video, virtual reality, motion tracking and mental training [15-17] In this study, we examine the effect of two neurofeedback protocols on the acquisition of microsurgical skills by a group of trainee ophthalmic surgeons. Specifically, fast wave training has been shown to facilitate sustained attention providing a relaxed attentional focus and increasing working memory [6-8], of particular importance for surgery which requires agility, concentration and stamina for long periods of time [18,19]. The sensory motor rhythm neurofeedback protocol helps relax the motor system which is vital in complex sensory-motor performance [4,11]. On the other hand slow wave training may benefit both stamina and morale, for aside from relaxation, this protocol enhances mood and well-being, through putative action on the limbic emotion system [10,31] Ophthalmic surgery, by virtue of the scale at which surgery is undertaken and the extreme adverse consequence of error, provides an ideal model with which to evaluate the potential benefits of neurofeedback. In brief, surgical performance in a skills laboratory [14] was assessed by means of two principal measures, surgical time and technique, representing the main critical dimensions in surgical proficiency: pace and accuracy [20,21]. Our initial hypothesis was that neurofeedback training would be able to successfully modify these measures with the aim of enhancing individual surgical skills-scheduled within the context of standardized and ongoing medical training – by modulating general cerebral function (via mechanisms of neuroplasticity) towards more 'efficacious' neural information processing appropriate to both the execution, as well as the retention, of fine sensorimotor maneuvers. In this regard surgeons were neither expected nor instructed to emulate or recollect 'neurofeedback conditions' on their own immediately prior to or during their performances, rather it was envisaged that the neuromodulatory effects of sustained control of the EEG would cumulate and be simultaneously active during the course of the multiple surgical training sessions as well as during the final performance.

The first protocol, known as SMR-Theta, aims to elevate the low beta "Sensorimotor Rhythm" [SMR] (12–15 Hz) while concurrently suppressing theta activity (4–7 Hz), and has been shown to enhance perceptual sensitivity and attentional performance in healthy subjects [4,6,7] resulting in decreased somatosensory and motor interference in basal ganglia/thalamocortical circuits [1,22]. This most likely occurs through the reinforcement of GABAergic inhibitory oscillations, such as those implicated in sensorimotor gating [23], the genesis of sleep spindles [24], and in reduction of seizure thresholds [25]. On the other hand, latest research points to a possible relationship between the SMR rhythm and long term potentiation (LTP), widely regarded as the main mechanism behind long term memory. For example, stimulating bursts of oscillations in this frequency range induced long-term modifications of excitatory neocortical synapses [26]. Moreover, 7–14 Hz spindling has also been proposed to 'open molecular gates of plasticity [27], by activating Ca^2^+ currents prior to transition to stage 1 sleep. This role in facilitating sensorimotor control and memory has clear implications for microsurgical performance. The second protocol, commonly referred to as Alpha-Theta (AT), aims to raise the ratio of theta (5–8 Hz) over alpha (8–11 Hz) activity levels during a wakeful eyes-closed condition in order to induce a deep relaxation state, given the association between theta activity and meditative states [28] and the wakefulness-to-sleep transition [29]. It has been especially employed as a complementary therapy in post-traumatic stress disorder (PTSD) [30], substance abuse [3], and has been shown to increase wellbeing in socially withdrawn students [10], as well as enhance artistry in music and dance performance [11,12]. Its impact on motivation and mood is thought to be mediated through limbic activation and its effects on creativity and sensorimotor performance mediated through its influence on long distance connectivity [31]. SMR-Theta feedback was visual with eyes open and included a 10 second break after 170 seconds, for a total of 8 such training 'periods', whereas ALPHA training was auditory and subjects were told to relax in an eyes closed condition, which was uninterrupted for a full 27 minutes.

## Results

One-way ANOVA disclosed no statistically significant differences between SMR, AT, and control groups in the number of days that elapsed between pre- and post-training assessments (F(2,25) = 0.34, p = 0.72). Furthermore, one-way ANOVAs confirmed that there were no statistically significant initial baseline differences between SMR, AT, and control groups in years of prior training (F(2,25) = 0.53, p = 0.59) or on initial baseline measures of surgical time [OVERALL: F(2,25) = 0.45, p = 0.64; TASK: F(2,25) = 0.48, p = 0.63; PAUSE: F(2,25) = 0.12, p = 0.89; SUTURE: F(2,25) = 0.85, p = 0.44], technique [(OVERALL: (F(2,25) = 0.52, p = 0.60; SUTURE: (F(2,25) = 0.13, p = 0.88], and anxiety [STATE: F(2,20) = 0.5, p = 0.61; TRAIT: F(2,20) = 0.78, p = 0.47]. Direct t-test comparisons between AT and SMR groups did not reveal significant differences between initial baseline measures of surgical time [TASK: t_18 _= -0.68, p = 0.51], or technique [(OVERALL: t_18 _= 0.92, p = 0.37]. Equally, there were no statistically significant initial baseline differences between EEG training ratios of median split SMR (F(1,79) = 0.77, p = 0.38) and AT (F(1,96) = 0.83, p = 0.37) groups according to higher and lower performance change scores in task time and overall technique, respectively. No significant differences were detected for initial baseline measures of surgical time or technique between low and high performers within SMR and AT groups respectively.

### Surgical Time

**Figure 1:**
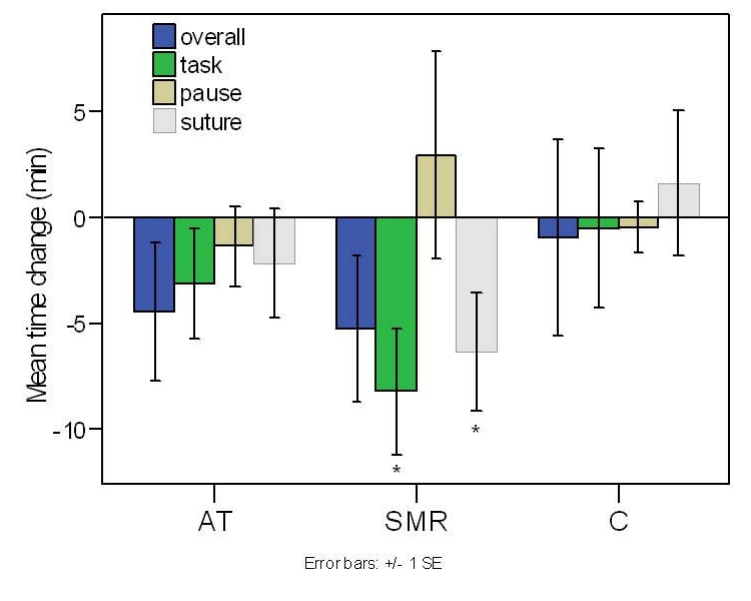
**Mean pre-post change in surgical performance time**. For Alpha-Theta (AT), SMR-Theta (SMR), and control (C) groups. In contrast to the control group, there is a significant reduction in total time on task as well as in the suture task for the SMR group. Marginal improvement is also seen in overall performance time for both SMR and AT groups. Error bars signify the standard error of the mean (SEM).

Results averaged across tasks are shown in Fig 1, together with the individual "suture" task. A TIME × GROUP repeated measures ANOVA disclosed in line with hypotheses a main effect of TIME for task time (F(1,25) = 4.92, p = 0.036), as seen in Fig 1. Paired t-tests confirmed that the 26% mean improvement (effect size d = 0.49) following SMR training differed significantly pre-post (8:41 min and 6:24 min: t_9 _= 2.80, p = 0.021), whereas the 12% mean improvement in the AT group was not significant (7:16 min and 6:24 min: t_9 _= 1.20, p = 0.26), in comparison to a negligible change in the control group (7:12 min and 7:04 min: t_7 _= 0.13, p = 0.90). Moreover, the SMR-group exhibited a significant decrease in the duration of the suture task (t_9 _= 2.26, p = 0.050). Regarding overall performance time, there was only a weak tendency for an improvement (SMR-group: t_9 _= 1.51, p = 0.083, one tailed; AT-group: t_9 _= 1.37, p = 0.10 one tailed; control group: t_7 _= 0.21, n.s.). There was no significant change in the mean pause time for any of the groups, although interestingly there was a non-significant average increase for the SMR-group (t_9 _= -0.61, p = 0.56). As can be seen from Table 1, the average baseline task time for the SMR group (8:41) was slightly higher, albeit non-significantly, than in the AT (7:16) and control (7:12) groups. At post assessment, both SMR (6:24) and AT (6:24) neurofeedback groups demonstrated a lower final time in comparison to the control (7:04) group, although the difference was not significant according to a one-way ANOVA (F(2,25) = 0.131, p = 0.878). A similar relationship was seen for the individual 'suture' task (Table 1). In summary there was a significant improvement in task time following SMR training, from a level that was non-significantly longer than in the other groups prior to training to a level comparable to the AT group following training. There were no significant changes in the AT and control groups. The reduction in task time in the SMR group was also paralleled by a reduction in the suture task, regarded as the most complex of the tasks.

**Table 1:** Mean pre and post values of surgical times and technique scores.

	AT		SMR		C	
	pre	post	pre	post	pre	post
Overall time (min)	09:26	08:12	11:00	09:32	09:14	08:58
s.d.	03:14	02:11	06:26	08:18	01:39	04:14
Task time	07:16	06:24	08:41	06:24	07:12	07:04
s.d.	02:18	01:47	05:36	03:37	01:43	03:39
Pause time (min)	02:10	01:48	02:19	03:08	02:02	01:54
s.d.	01:36	00:30	01:11	04:48	00:33	00:46
Suture time (min)	03:33	02:57	04:45	03:00	03:20	03:48
s.d.	01:52	00:59	03:32	01:47	01:34	03:24
Overall technique (%)	82.2	84.0	79.6	83.7	81.8	81.6
s.d.	5.7	4.1	7.3	6.2	5.6	4.9
Suture technique (%)	78.1	79.4	75.0	81.5	77.8	77.7
s.d.	12.7	8.7	9.2	5.3	10.1	6.7

Alpha-Theta (AT), SMR-Theta (SMR), and control (C) groups and standard deviations (s.d.)

### Surgical Technique

Inter-rater reliability analysis with the intraclass correlation coefficient (ICC) between judges' overall scores disclosed satisfactory concordance for all three groups (ICC_55 _= 0.63), and especially for the SMR group (ICC_19 _= 0.85).

**Figure 2:**
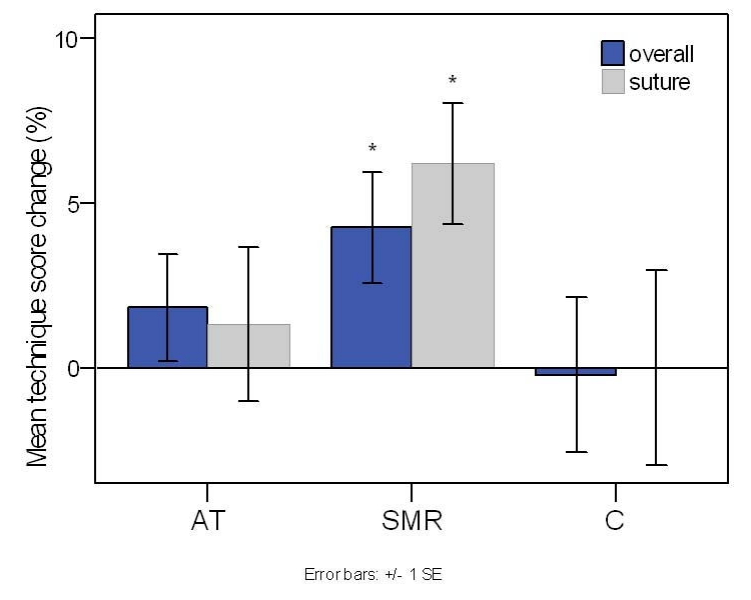
**Mean pre-scores minus mean post-scores in surgical technique**. For Alpha-Theta (AT), SMR-Theta (SMR), and control (C) groups. In contrast to the control group, there is a significant increase in judges ratings' of both overall technique and particularly in the more difficult suture task for the SMR group. Marginal improvements are seen for the AT group although they are accompanied by large standard errors. Error bars signify the standard error of the mean (SEM).

Average group results are shown in Fig 2. In keeping with the objective tests the SMR-group showed a reliable improvement from an overall score of 79.6% to 83.7% (z_10 _= -2.2, p = 0.028), equivalent to an effect size of d = 0.62. Specifically for the SMR-group, there was a significant change in the suture task (z_10 _= -2.38, p = 0.018), with d = 0.87. In fact, subjective and objective performance measures were interrelated in so far as improvements in overall technique were correlated with reductions in overall task time for the SMR-group (rho = -0.70, p = 0.036). Regarding the AT protocol, while positive mean changes were exhibited overall (from 82.2% to 84%), the difference did not prove statistically significant. As seen in Table 1, the initial overall technique score for the SMR group (79.6) was somewhat, although not statistically, lower, than in the AT (82.2) and control (81.8) groups. Subsequently, both SMR (83.7) and AT (84.0) experimental groups demonstrated a higher final score in comparison to the control (81.6) group, although this difference was not significant in one-way ANOVA (F(2,25) = 0.55, p = 0.58). A parallel trend was observed for the technique rating on the 'suture' task (Table 1).

### Neurofeedback learning and surgical performance relationships

The mean group results disclosed evidence of learning both within and between sessions. The whole neurofeedback session was divided into 3-minute time blocks called 'periods', starting with period 1 which was the feedback-free baseline. Periods 2 and 9 denote the start and end of neurofeedback, respectively. The within session SMR/theta ratio increased from baseline to period 9 (rho = 0.08, p = 0.035), underpinned by reductions in theta (paired t_58 _= 2.27, p < 0.03). The AT ratios increased reaching a plateau at period 5 (paired t_77 _= 3.9, p < 0.01). Across session learning reached a peak with session 5 for the SMR-group (rho = 0.114, p = 0.017; paired t_83 _= -3.84, p < 0.01) and with session 7 for the AT-group (rho = 0.73, p = 0.06; paired t_83 _= -3.66, p < 0.01).

With the SMR-group, there was a tendency for successful within-session SMR-training to be associated with an increase in total pause time (R = 0.584, p = 0.077), while the latter correlated with a lower task time (R = -0.72, p = 0.019), suggesting a more modulated performance. Although AT training did not produce significant improvements for the group as a whole, successful within-session AT training correlated significantly with overall improvement in surgical technique (R = 0.638, p = 0.047).

**Figure 3:**
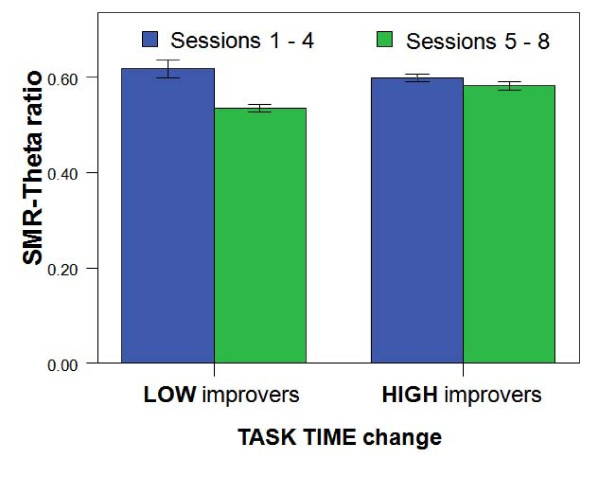
**Mean SMR/Theta ratio between 1st/2nd halves of neurofeedback sessions (1–4 and 5–8), as an index of incremental learning; for high vs low improvers in surgical task time in the SMR group**. Error bars signify the standard error of the mean (SEM).

The positive relation between SMR learning and improvement in surgical performance was elucidated further with the SMR group (with a total of 10 subjects) being median split into two equal halves of 5 subjects, according to each subject's absolute *change *in surgical task time. The top 5 subjects in the group that had the largest *reductions *in task time were designated as 'high' improvers, whereas the bottom 5 were labeled 'low' improvers. Mean percentage change of the SMR/theta ratio was then computed for each subgroup between the first half (1–4) and second half (5–8) of the total sessions. There was a Group × Ratio interaction (F(1,79) = 7.4, p < 0.01), whereby a 10% highly significant reduction (-0.08, SD 0.15; p < 0.01) in the learning ratio in low improvers in the second half which was not found in high improvers (-0.02, SD 0.03; n.s.). This falloff in learning ratio in low improvers was elucidated by examining absolute EEG bands separately. As shown in Fig 3, there was an interaction between Group × Theta amplitude change (F(1,79) = 5.9, p = 0.017) whereby the theta amplitude significantly *increased *in the low improvers in the second half of sessions (0.60, SD 1.2) compared with a non-significant decrease in high improvers (-0.48, SD 2.5). Of relevance to the falloff in training in low improvers, there was also a significantly greater number of days (8.5 to 4.8, d = 0.76) elapsed between the latter half of training sessions of *low *versus *high *improvers (unpaired t_34 _= -2.2, p = 0.035); implying that the longer the intersession interval, the poorer the learning in the direction of training goals.

Finally, the same investigative approach when applied to the AT group, disclosed a reliable positive change in the EEG ratio when median split according to surgical technique (Group × Ratio; F(1,96) = -5.2, p = 0.025) in higher versus lower improvers (0.05, SD 0.10; -0.002, SD 0.13) respectively.

### Anxiety Scale

**Figure 4:**
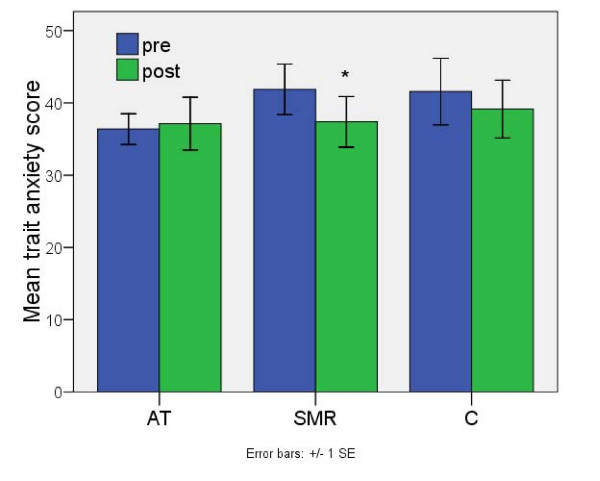
**Mean pre-scores minus mean post-scores in trait anxiety**. For Alpha-Theta (AT), SMR-Theta (SMR), and control (C) groups. There is a significant decrease in trait anxiety (i.e. in the past few weeks) score for the SMR group. No reliable changes are present in either the control or AT groups. Error bars signify the standard error of the mean (SEM).

The Spielberger Anxiety Inventory revealed a significant reduction in trait anxiety for the SMR group (d = 0.46) compared to the controls (SMR 41.9 to 37.4, z8 = 2.38, p = 0.017; AT 36.4 to 37.1, n.s.; controls 41.6 to 39.1, n.s) as seen in Fig 4. There were no reliable changes in state anxiety, however, when the relation between pre-post anxiety change and EEG training was investigated, a correlation between state anxiety change and AT within-session learning (R = -0.66, p = 0.053) was observed.

## Discussion

Benefits were achieved by trainee opthalmic surgeons in a skills lab simulation of a cataract operation as a result of eight half hour sessions of EEG-neurofeedback training. In particular for the SMR-Theta group there was a 26% reduction in the total time to perform the tasks (Fig 1), while the most significant change occurred during the more complex of the skills, involving tying a suture following an incision in the artificial cornea. Of the four tasks monitored this is the one that took the longest to complete and the one with the most reported difficulty. The objective timed measure of improvement was corroborated by the subjective ratings of the two consultant ophthalmic surgeons on an exam scale of surgical technique (Fig 2). Additionally, there was on average an increased pause time between tasks. This 'time for preparation' was positively correlated with reduced task time, indicating a more modulated performance – an increased preparation time coupled with reduced task times. The significant reduction in self-reported anxiety (Fig 3) over the preceding weeks would also be compatible with achieving a more modulated performance.

Before considering the results further the experimental limitations of the study should be acknowledged.

In light of the small group sizes ANOVAs on surgical measures did not yield significant interactions between experimental and control protocols, mainly resulting from irregular group variances. Moreover, longer term follow-up tests were not conducted in this study, mainly because the rotational system of the National Health Service does not grant trainee surgeons a permanent locus. These limitations could be minimised in future with greater numbers of subjects and with surgeons of more fixed positions.

Random allocation of subjects led to a slightly uneven, albeit not statistically significant (verified by one-way ANOVA), distribution of initial performance measures between groups for both task time and overall technique. There was an initial preferential performance to the AT and control groups, leading to the possibility that their ability to improve as had occurred with SMR training was compromised. However, regarding time on task, as can be seen in Table 1, a mean improvement, albeit non-significant was seen in the AT group. Furthermore regarding technique scores, participants' scores fell far short of a ceiling effect of 100%.

We also performed analyses on the final (post) group values of task time and overall technique. As seen in Table 1, although both SMR and AT groups had somewhat lower task time and higher overall technique at post assessment compared to the control group, no significant differences were revealed between groups with one-way ANOVAs. With small sample sizes, there is again the ever-present danger of the 'regression to the mean' effect. Also, given our a priori hypotheses regarding surgical improvement, we did not perform a correction for multiple comparisons; however the latter would be of greater concern in cases where variables exhibit greater independence (in contrast, intercorrelations between surgical variables was high). As described below, the manifest associations between task time change and neurofeedback training indices (training ratio, session frequency) in the SMR group render random effects of this type less likely.

Regarding the subjective ratings of surgical technique (in contrast to the objective timed measures) the total inter-rater reliability of 0.64 is not high enough to establish an explicit conclusion regarding the effects on technique for all the three groups. However, the protocol with the more promising results for surgical training, the SMR protocol, demonstrated a good reliability of 0.84, indicating a clearly positive effect for this group.

One may question the strength of the control protocol given the wait-list design, as the same subjects were used for the control and training assessments, which inevitably leaves room for practice or time effects. However, with regard to practice effects the training change was analysed strictly between back-to-back assessments (for the wait-list control group the second and third assessments), so differences that occurred during the previous control period were entirely excluded. Furthermore, surplus analyses carried out for changes between first and final assessments of the control group on all variables did not produce any disparate conclusions. Test-retest interval times also indicated comparable elapsed time among control and training group assessments.

While comparing two intervention conditions with a no-intervention control group, the issue of therapist contact or 'placebo' effects may be raised. However, taking into consideration the different impact of the two experimental protocols on performance, it seems unlikely that placebo should account for the observed outcome. Nevertheless, in order to completely rule out the possibility of placebo, future work would benefit from including a 'sham' neurofeedback control group, instead of a wait-list.

One of the practical difficulties in running the study was the availability of the trainee surgeons to fit the neurofeedback sessions into their busy schedule. Some trainees had undesirably long intersession intervals of more than a week which may have led to suboptimal conditions for learning. Indeed in the SMR group those who improved the most in reducing task time attended on a bi-weekly basis. The practical limitations may have affected training with the alternative AT protocol which aims to induce a hypnagogic state and which has demonstrable benefits for peak performance training [31]. There was a falling off in the theta/alpha ratio during the last six minutes of the session, as if in anticipation of the session end and the next diary commitment. Notwithstanding, while there were no significant advantages for the AT group as a whole, learned control of the theta/alpha ratio within sessions correlated positively with ratings of surgical technique, and the median split subdivision based on improvement in technique disclosed that those improving most were the more successful in increasing their theta/alpha ratio between sessions. Within session learning was also positively correlated with reduction in state anxiety.

However, the most significant benefits of neurofeedback training were obtained by elevating the low beta sensorimotor rhythm band (12–15 Hz) while simultaneously inhibiting slow wave theta (4–7 Hz) activity. The reliability of the effects with this protocol is strengthened by the finding that those participants who showed the greater improvement in time on task were those who showed increased maintenance of the SMR/theta ratio *as well as *a lower number of days between neurofeedback training sessions. The development of surgical skill could be regarded as jointly evolving on levels of both central executive control (attention) and the retention of strict motor procedures. In accordance with this model, our results remain in line with previous research on SMR-Theta training as a viable method for enhancing executive attention [6,7], and are further supported by an fMRI study [32] reporting plastic changes following training with this neurofeedback protocol in anterior cingulate (part of the attentional network) as well as caudate nucleus and substantia nigra, central components of motor basal ganglia function. Synchronization of the SMR low beta rhythm has been found to be directly implicated in motor response inhibition during a GO/NO-GO task [33]. Moreover, recent evidence indicates that elevated SMR activity predicts performance improvements on procedural motor tasks and is related to consolidation of motor tasks following sleep [34,35], while a latest report suggests direct increases in sleep spindle power following SMR neurofeedback are associated with individual enhancement of memory [36]. In light of the previously cited studies linking SMR, sleep spindle activity, and synaptic plasticity [24,26,27], it is possible that SMR neurofeedback may have aided the priming and/or preservation of new synaptic connections [37] either before they were consolidated during sleep, or by directly influencing the sleep EEG parameters [36] which putatively modulate the memory mechanisms inherent to sleep [26,27].

In addition, the protocol required the suppression of *theta *(4–7 Hz) amplitude, whose training reduction while significant for the group as a whole, in later sessions became uniquely elevated in the *low *improvers. Decrease in cortical theta power has been reported during activation of the attentional alerting network [38], found to predict better reaction time performance [39], and interpreted as a stronger inhibition of long-term memory networks aiding the processing of external stimuli. Moreover, desynchronisation of cortical slow waves, which include the theta range, is an indicator of increased cortical activation, elicited for instance by stimulation of the cholinergic or noradrenergic systems [40]. An attractive and tentative explanation might combine the latter with the large body of evidence that pharmacological activation of these systems leads to robust enhancement of LTP and practice-dependent motor learning (for review see [41]). New support for this interpretation has emerged from our recent experiment (in preparation) demonstrating that reductions in alpha and theta power post-neurofeedback coincide with increased corticospinal excitability when assessed with TMS (transcranial magnetic stimulation). This would support a more modern yet complementary hypothesis of synaptic potentiation occurrence during wakefulness, which has been demonstrated to be causally related to cellular changes dependent on noradrenergic release, such as the induction of LTP-related genes [42].

## Conclusion

In conclusion, and to the best of our knowledge, this is the first study to show extensive evidence for performance enhancement in microsurgical procedure by means of EEG self-regulation. More specifically, our data have shown that SMR/theta training provides statistically reliable improvements in surgical technique, together with a 26% reduction in time on task. This may lead to reductions in surgical stress, contact time with the eye, and lessen the risk and extent of surgical complications [43], thereby improving surgical outcomes. Our results are further supported by good agreements between qualitative and quantitative performance assessment measures.

## Methods

The participants were 22 National Health Service trainee ophthalmic surgeons (10 males, 12 females; mean age 33.5, SD 5.12) from the Western Eye Hospital, London, UK. Following approval by the College Research Ethics Committee, written informed consent was obtained from all volunteers and they did not receive any monetary reward. Subjects were allocated at random to one of 2 training groups: SMR-Theta neurofeedback (n = 10) or Alpha-Theta neurofeedback (n = 10). Both groups underwent 8 half hour sessions of training, with the assigned protocol, during a period of 2–3 months of a nationally standardized surgical training curriculum. Average session frequency per subject was calculated from the number of days elapsed between sessions. A randomly selected subset in each protocol group (SMR n = 4, AT n = 4) was assigned to a third group, a wait-list 'control' group (n = 8), in order to control for the effects of practice and time during surgical training. These subjects undertook an additional surgical assessment which occurred about three months prior to the start of neurofeedback training, otherwise they received no prior intervention apart from the standard ongoing surgical training curriculum. Subsequently they completed the same experimental procedure as their respective training group. Two of the wait-list subjects did not complete their neurofeedback training, so that only their control assessments were included in the analysis. On each surgical assessment subjects firstly completed a self-report Spielberger's State and Trait Anxiety Inventory [44], consisting of 20 questions on a four-point Likert scale with separate scores of state and trait anxiety, respectively defined as anxiety felt at the moment and in the past week.

### Surgical measures

The surgical performance assessment consisted of four sub-tasks, each a simulation of part of a cataract operation using a model eye and completed in the following order: 'sideport incision', 'phaco wound', 'capsulorrhexis' and 'suture'. Current ophthalmic surgical practice involves extra manipulation of the eye through a small self-sealing wound in the cornea ('sideport incision'). Removal of the cataractous lens is carried out by ('phaco') emulsification using minimally invasive ultrasound energy transmitted via a probe inserted through a self-sealing corneal wound ('phaco wound'). Prior to phacoemulsification a round hole is made in the front coating of the lens ('capsulorrhexis). A stitch ('suture') is sometimes used for extra wound security at the end of the operation. These simulated surgical procedures were performed in standardized conditions. Surgical performance was recorded on digital video from two angles (surgical microscope view and position of fingers from the side), and then scored by two expert judges. Both judges were consultant ophthalmic surgeons as well as experienced teachers, and were masked to individual identity, group membership and performance order. They rated the discrete surgical tasks individually, with the same score template structured in subsections, on a two-point scale for 54 criteria fulfilled or unfulfilled (e.g. correct angle of blade parallel to iris). The chronometer on each video recording was used to record objectively the time to completion of the surgical tasks. Overall time was defined as the start to finish time. This was mathematically equivalent to the sum of the task time and pause time, the former being defined as time spent on task (from the first contact with the eye), while the latter defined as the time spent between tasks in preparation.

### SMR-Theta training

Training began with a 3-min baseline period during which the EEG-band amplitudes were recorded at rest with eyes open, in the absence of feedback. This baseline was then used as the initial criterion for the contingent feedback that followed. This consisted of eight 170 s feedback periods, with 10 s breaks in between them. Band amplitude values are transformed online into geometrical visual feedback representations, displayed on a 15" computer monitor. In the "Space Race" game for example, the speed at which 3 parallel spaceships move in relation to each other is dependent on the 3 respective brainwave amplitudes being trained. Subjects learn to associate the varying conditions during the game with their brain EEG parameters. This learning process may or may not be consciously driven, and is referred to as operant conditioning. Operant contingencies were such that rewards (or 'points') were gained whenever the subject increased SMR band activity without concurrent increases in theta (4–7 Hz) and high beta (22–30 Hz) band activity. The subjects were seated in a comfortable chair about 1.5 m from the monitor and they were instructed to simply let the feedback process guide them into learning how to maximize their point score. The feedback thresholds were automatically reset during each break period to maintain a constant level of reinforcement. The reward band threshold was set at 0.8 times its baseline average, while the high beta and theta inhibit thresholds were set at 1.2 times their baseline average. There was no form of point-based negative feedback, although an EMG inhibit (40–70 Hz) band ensured that subjects would not be rewarded for simply tensing cranial muscles. All neurofeedback EEG was recorded from the motor cortex (Cz), with reference and ground electrodes placed on either earlobe; impedance was kept below 10 kΩ.

### Alpha-Theta training

The alpha-theta protocol involved only auditory feedback with eyes closed. A 3-min eyes-closed baseline was first recorded in the absence of feedback, this was then used to set initial alpha and theta band thresholds. Subsequently, eyes-closed auditory feedback was engaged for a continuous 27 minutes. Both alpha and theta band related sounds acted as rewards and were intended to induce relaxation. Alpha activity was represented by a 'babbling brook' background sound and theta by an 'ocean waves' sound, the latter was set to have a higher priority over the former when both reward conditions were met. The operant contingencies were by this means intended to induce higher theta-to-alpha ratios under waking conditions. Trains of suprathreshold alpha and theta activity elicited a high and low pitch gong sound respectively. Subjects wore a set of headphones and relaxed in a comfortable reclining chair. They were instructed to relax deeply in order to achieve an increase in the amount of theta sound representation, but to avoid falling asleep. A delta band (1–4 Hz) EEG inhibit was also implemented to preclude the latter.

During the course of the session the experimenter aimed to maintain alpha and theta reward band values within a range of minimally 30% to maximally 65% of time above threshold. The EEG was recorded from parietal lobe (Pz), with reference and ground electrodes placed on either earlobe, impedance was kept below 10 kΩ.

### Apparatus and EEG analysis

EEG signals were registered using a Procomp+ differential amplifier (Thought Technology Ltd, Montreal, QC), and neurofeedback training was carried out with Neurocybernetics EEGer software (Encino, CA). EEG was sampled at 160 Hz by the A/D converter in the Procomp+ and FFT (Fast Fourier Transform) bandpass filtered to extract high beta (22–30 Hz), SMR (12–15 Hz), alpha (8–11 Hz) and theta (4–7 Hz) amplitudes in microVolts, with a smoothing time constant of 0.5 seconds. A low pass filter was additionally used at 50 Hz. No other classification of signal is performed. Following Lubar et al. [2] successful neurofeedback learning was defined by an increase in the training ratio, or the ratio of activity in the training frequency relative to the inhibitory frequencies. For Alpha-Theta training this was expressed by theta divided by alpha amplitudes in microVolts, or the theta/alpha ratio, and for SMR training by the SMR/theta ratio. Two additional indices of neurofeedback learning were calculated for each protocol to establish relationships of EEG learning across time, a within-session learning coefficient (the correlation between the mean training ratio of each 3-min period and the number of periods) and an across-session coefficient (the correlation between the mean training ratio of each session and the session number).

### Statistical Analyses

Pre- versus post-training outcome of the wait-list group was calculated between latter assessments (numbers 2 and 3), whereby any changes occurring during the control period were discounted. Effects on performance time were assessed by a Group × Time (3 × 2) repeated measures ANOVA. Paired t-tests or Wilcoxon tests were respectively carried out on time (parametric) and technique (non-parametric) measures in order to examine pre-post changes. For groups where significant performance effects were detected, the relation between the learning indices of each neurofeedback protocol and performance change score (subtracting post-training from pre-training values) was analyzed by means of correlation analysis. Lastly, performers' mean EEG training ratios were also compared via a median split of higher versus lower performance change scores.

## Authors' contributions

TR contributed to the neurofeedback training, follow-up recordings, data analysis and wrote the manuscript with JHG. PAB, LB, MJM and JHG contributed to the design and conception of the study. PAB and LB scored the surgical video performances. LAP initially instructed TR on the neurofeedback training protocols. All authors read and approved the final manuscript.
